# Supplementary material for: A novel sorting signal for RNA packaging into small extracellular vesicles
Source: Sci Rep. 2023 Oct 13;13:17436. doi: 10.1038/s41598-023-44218-z (PMC10575923; doi:10.1038/s41598-023-44218-z)
Supplement: Supplementary file 1 — Supplementary Information. [file 41598_2023_44218_MOESM1_ESM.pdf]

Supplementary Information

A novel sorting signal for RNA packaging into small extracellular vesicles

Yuma Oka[1*], Kosei Tanaka[1], Yuki Kawasaki[1*]

[1]H.U. Group Research Institute G.K., Fuchigami 50, Akiruno, Tokyo, Japan

*Co-corresponding authors

E-mail: yuma.oka@hugp.com, yuki.kawasaki@hugp.com

Supplementary Figures and Tables

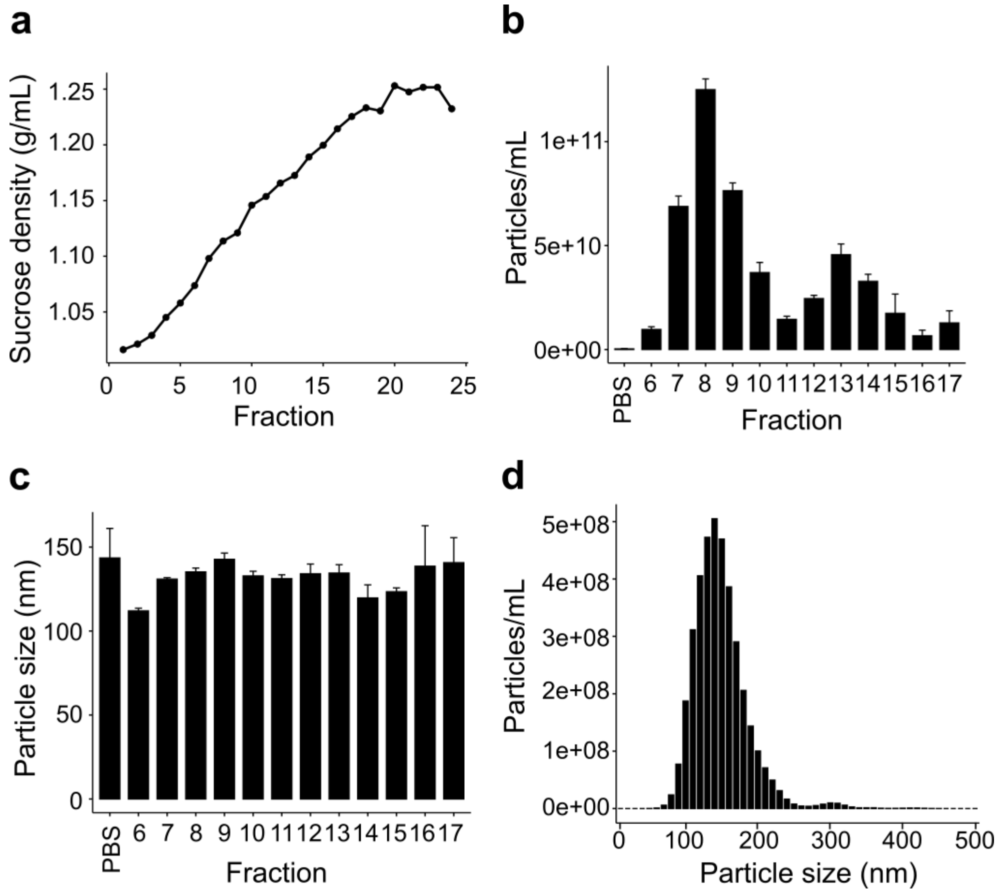

Fig. S1 Fractions obtained through sucrose density gradient ultracentrifugation. (a) Sucrose densities of each fraction. (b) Particle numbers in each fraction as measured by nanoparticle tracking analysis. Error bars are shown as ±standard error. (c) Average particle size in each fraction as measured by nanoparticle tracking analysis. Error bars are shown as ±standard error. (d) Histogram of particle size distribution of fraction 9.

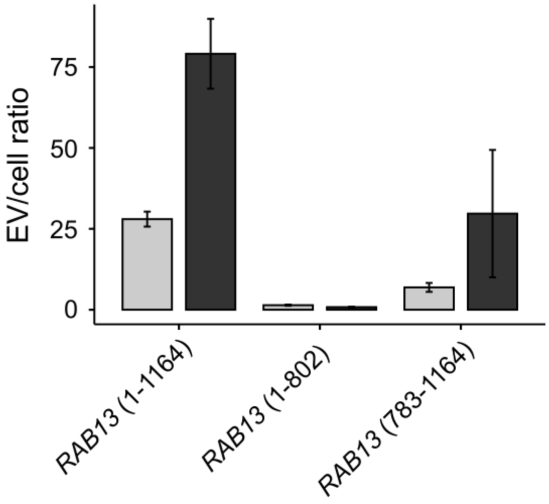

Fig. S2 Enrichment level of *RAB13* complete or partial sequences in H1299 and MCF7 cell lines. The transcripts of interest in CD9 positive EVs and cells were quantified and normalized to *GAPDH*, expressed as a ratio of transcript level in CD9 positive EVs to that in cells. Gray: H1299, Black: MCF7.

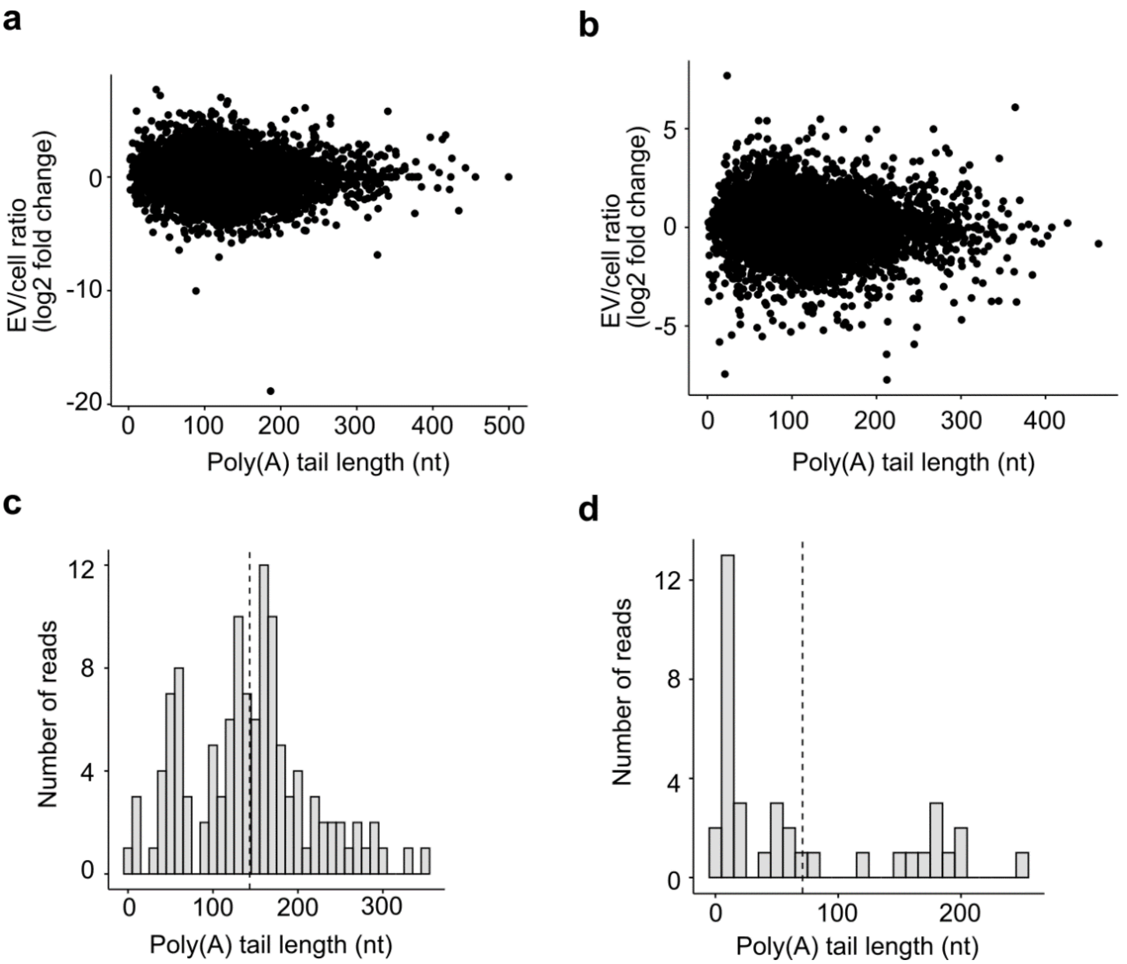

Fig. S3 Association of poly(A) tail length with EV enrichment level. (a) Scatter plot showing the correlation between endogenous genes' poly(A) tail length and enrichment level within CD9 positive EVs. Poly(A) tail length data was obtained from HEK293 cells expressing *eGFP* fusion gene containing the sense or (b) scrambled *RAB13* (783-1164) sequence. (c) Poly(A) tail length distribution for the transgene extracted from cells expressing *eGFP* fusion gene containing the sense or (d) scrambled *RAB13* (783-1164) sequence.

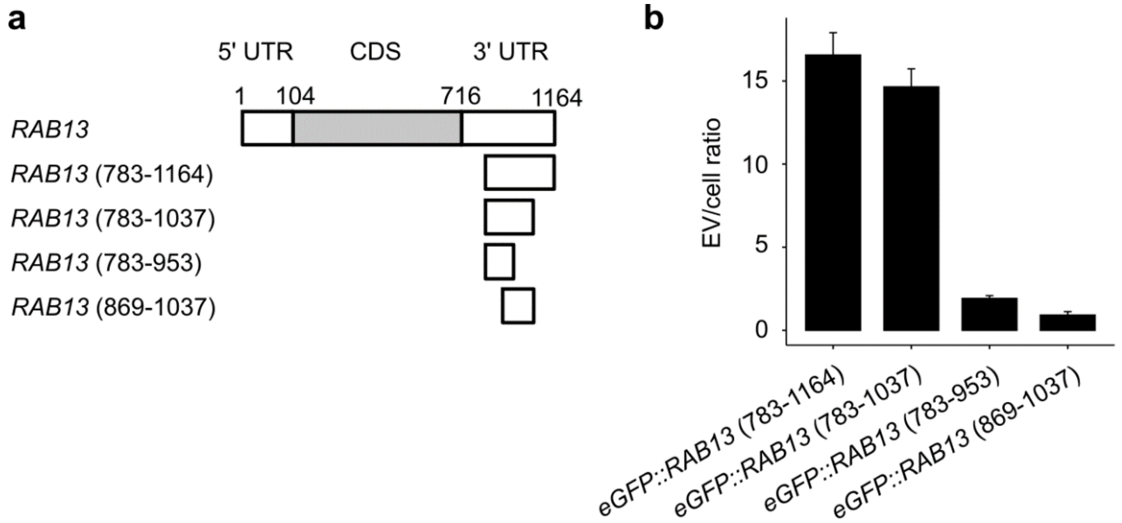

Fig. S4. *RAB13* mRNA enrichment level of transgenes fused with *RAB13* 3'UTR subsequence in CD9 positive EVs. (a) Diagram of the *RAB13* mRNA and its partial sequences. The indicated number in parentheses corresponds to the sequence positions of *RAB13*. (b) Enrichment level of transgenes in the CD9 positive EVs from HEK293 cells expressing the partial sequences of *RAB13* fused with *eGFP*.

a

b

c

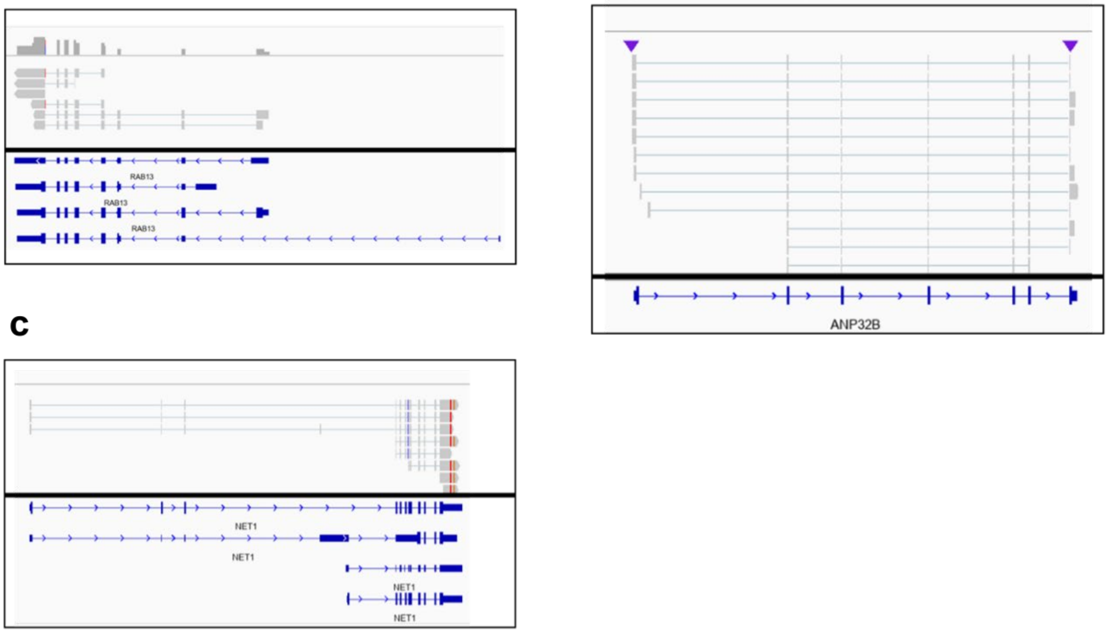

Fig. S5. Full-length reads of enriched transcripts detected in CD9 positive EVs. Mapped reads to (a) *RAB13*, (b) *ANP32B*, and (c) *NET1* reference sequences were visualized using Integrated Genome Viewer.

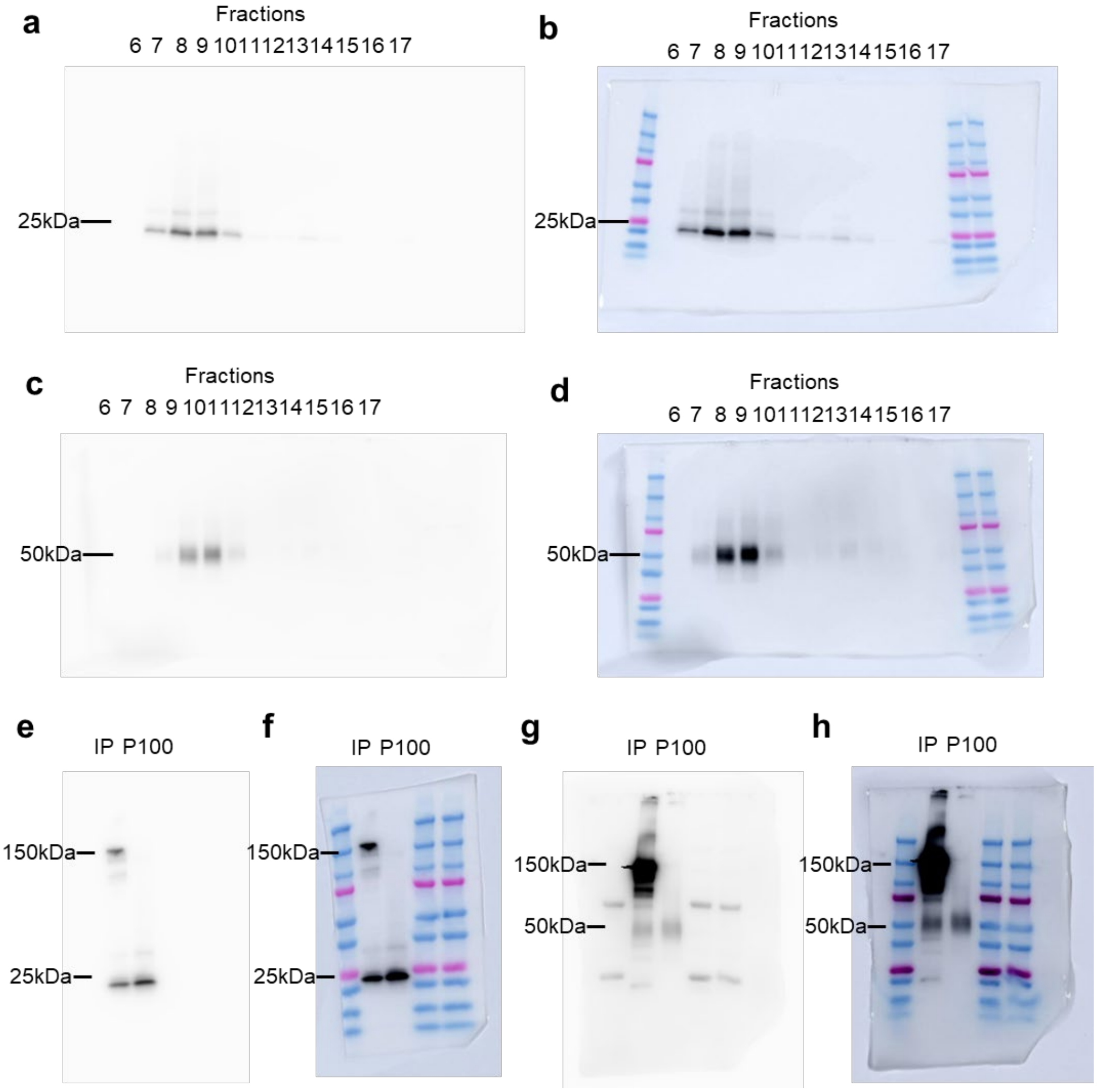

Fig. S6. Raw western blot images. Two images are presented for each dataset: one with the uncropped original signal (a, c, e, g) and the other with a molecular weight ladder added (b, d, f, h). CD9 is shown in (a) and (b), and CD63 is shown in (c) and (d) for Fig. 1a. CD9 is shown in (e) and (f), and CD63 is shown in (g) and (h) for Fig. 1b. Note that the upper bands around 150 kDa of IP samples in (e) to (h) correspond to the magnetic beads with anti-CD9 antibody.

Table S1. Housekeeping genes and enriched genes in RNA sequencing analysis

Housekeeping or Enriched	Gene	EV FPKM	Cell FPKM	Log2(fold change)	P-value
Housekeeping	*GAPDH*	2509.12	966.172	1.37683	5.00E-05
Housekeeping	*ACTG1*	5989.05	6487.66	-0.115371	0.5884
Enriched	*RAB13*	15849.1	76.875	7.68767	5.00E-05
Enriched	*LUC7L3*	6675.59	98.4673	6.08311	5.00E-05
Enriched	*ANP32B*	2950.56	69.8478	5.40063	5.00E-05
Enriched	*NET1*	1064.41	25.0559	5.40876	5.00E-05
Enriched	*MTURN*	844.094	19.2636	5.45345	5.00E-05
Enriched	*HMGN5*	773.797	17.2521	5.48711	5.00E-05
Enriched	*C22orf46*	658.727	13.6279	5.59505	5.00E-05
Enriched	*PLEKHA4*	646.602	5.04757	7.00115	5.00E-05
Enriched	*UPF2*	539.692	14.2281	5.24532	5.00E-05
Enriched	*CEP112*	231.148	3.43673	6.07163	5.00E-05
Enriched	*PGPEP1*	178.025	1.74012	6.67675	5.00E-05
Enriched	*C19orf68*	124.48	2.23571	5.79904	5.00E-05
Enriched	*CA11*	105.539	3.23839	5.02636	0.00035

Table S2. RT-qPCR Ct values

Fig.	Sample	Target gene	Ct	Ct	Ct
Fig. 1e	Fraction 1	*RAB13*	29.010	29.459	30.713
Fig. 1e	Fraction 2	*RAB13*	28.366	29.965	30.241
Fig. 1e	Fraction 3	*RAB13*	33.435	30.236	31.709
Fig. 1e	Fraction 4	*RAB13*	29.378	29.693	32.540
Fig. 1e	Fraction 5	*RAB13*	31.459	34.515	30.400
Fig. 1e	Fraction 6	*RAB13*	31.298	31.201	30.859
Fig. 1e	Fraction 7	*RAB13*	28.272	28.548	28.922
Fig. 1e	Fraction 8	*RAB13*	28.465	28.791	28.163
Fig. 1e	Fraction 9	*RAB13*	25.887	26.004	26.164
Fig. 1e	Fraction 10	*RAB13*	32.198	31.527	30.550
Fig. 1e	Fraction 11	*RAB13*	29.929	32.518	31.316
Fig. 1e	Fraction 12	*RAB13*	32.866	31.842	31.941
Fig. 1e	Fraction 13	*RAB13*	30.009	30.488	32.509
Fig. 1e	Fraction 14	*RAB13*	31.925	35.580	30.750
Fig. 1e	Fraction 15	*RAB13*	34.468	33.645	32.931
Fig. 1e	Fraction 16	*RAB13*	38.494	32.110	32.138
Fig. 1e	Fraction 17	*RAB13*	32.998	30.892	31.225
Fig. 1e	Fraction 18	*RAB13*	33.174	34.329	31.742
Fig. 1e	Fraction 19	*RAB13*	32.847	31.267	31.199
Fig. 1e	Fraction 20	*RAB13*	33.096	30.801	32.337
Fig. 1e	Fraction 21	*RAB13*	31.675	32.655	31.887
Fig. 1e	Fraction 22	*RAB13*	34.237	30.537	35.485
Fig. 1e	Fraction 23	*RAB13*	33.785	32.545	31.128
Fig. 1e	Fraction 24	*RAB13*	30.674	30.861	37.640

Fig. 1f	non-treatment	*PMMoV*	23.295	23.333	23.392
Fig. 1f	non-treatment	*RAB13*	33.501	32.325	33.803
Fig. 1f	RNase treatment	*PMMoV*	34.894	37.120	34.431
Fig. 1f	RNase treatment	*RAB13*	33.739	33.323	36.631

Table S3. Antibodies

Antibodies	Usage	Source	IDs
Mouse monoclonal anti-CD9	Immunoaffinity purification of sEVs	In-house	-
Mouse monoclonal anti-CD9	Western blot	CosmoBio	SHI-EXO-M01
Mouse monoclonal anti-CD63	Western blot	In-house	-
Rabbit Anti-Mouse Immunoglobulins/HRP	Western blot	Dako	P0260

Table S4. Primers and sequences

Primers and sequences	Sequence (5' → 3')	Description
RAB13 Fw	AGACAATAACTACTGCCTACTACCGTG	For quantifying endogenous *RAB13* (Fig. 1d-f)
RAB13 Rv	GAGCACTTGTTGGTGTTCTTCTTGTCA	
ANP32B Fw	GTCAGTGAGGAGGAAGAAGAATTTGG	For quantifying endogenous *ANP32B* (Fig. 1d)
ANP32B Rv	GTAGATTACCAAGAGGGACTACATGG	
NET1 Fw	CAGTCCAAGAGCTAGTCCTAGAAGAG	For quantifying endogenous *NET1* (Fig. 1d)
NET1 Rv	CTTCTACCTGAGTAACACTGGAAACTG	
LUC7L3 Fw	CAGAGAACAAGATAGAAAATCCAAGG	For quantifying endogenous *LUC7L3* (Fig. 1d)
LUC7L3 Rv	AACAGTAACAAAAAGCCCTAATCAAA	
ACTG1 Fw	CTTCCTTCCTGGGTATGGAATCTTG	For quantifying endogenous *ACTG1* (Fig. 1d)
ACTG1 Rv	CAAATTTCTATTCTCAATTAACCCATG	
PMMoV Fw	GAGTGGTTTGACCTTAACGTTTGA	For quantifying spike-in RNA (Fig. 1f)
PMMoV Rv	TTGTCGGTTGCAATGCAAGT	
pcDNA3.1 Fw	GAGCTCTCTGGCTAACTAGAGAACCC	For quantifying partial sequences of *RAB13* (Fig. 2b, S2)
pcDNA3.1 Rv	CAGACAATGCGATGCAATTTCCTC	
GAPDH Fw	ACTTTGTCAAGCTCATTTCCTGGTATGAC	For quantifying *GAPDH* as internal control (Fig. 1, 2, S2, S4)
GAPDH Rv	GGTACTTTATTGATGGTACATGACAAGGTG	
eGFP Fw	GACTGGGTGCTCAGGTAGTG	For quantifying fusion genes (Fig. 2c, S4)
eGFP Rv	CAAGATCCGCCACAACATCG	
PMMoV spike-in sequence	GCAGCAAAGGUAAUGGUAGCUGUGGUUU CAAAUGAGAGUGGUUUGACCUUAACGUU	For Spike-in control (Fig. 1f)

	UGAGAGGCCUACCGAAGCAAAUGUCGCAC<br>UUGCAUUGCAACCGACAAUUACAUCAAAG<br>GAGGAA

Supplementary Methods

Nanoparticle tracking analysis

Fractions obtained through sucrose density gradient ultracentrifugation and fractionation were diluted at 20-fold or 40-fold and subjected to nanoparticle tracking analysis using a NanoSight LM10 (Malvern Panalytical). The results were evaluated using Nanoparticle Tracking Analysis software (3.1).

Full length RNA sequencing and analysis

The conditioned medium from HEK293 cells was centrifuged at 2,000 × g for 5 min at 4 °C, followed by filtration through a 0.45 μm filter. The samples were enriched using an AMICON ULTRA-15 100 KDa cutoff (Merck Millipore) and resuspended in PBS. Subsequently, the enriched samples were cautiously layered onto 30 w/v% sucrose solution present in ultracentrifugation tubes and subjected to ultracentrifugation at 100,000 × g for 3 h at 4 °C. The collected sucrose solutions were mixed with PBS and underwent ultracentrifugation at 100,000 × g for 1 h for washing purposes. Total RNA was extracted using TRIzol LS Reagent (Thermo Fisher Scientific) following the manufacturer's instructions.

Library preparation and sequencing were performed according to Procedure-Checklist-Iso-Seq-Express-Template-Preparation-for-Sequel-and-Sequel-II-Systems version02 (PacBio). Briefly, cDNA synthesis and amplification were carried out using the NEBNext Single

Cell/Low Input cDNA Synthesis & Amplification Module (NEB), with primers from the Iso-Seq Express Oligo Kit (PacBio). The PCR cycle consisted of 20 cycles. The amplified cDNA was size-selected to remove fragments less than 1 kb using the ProNex® Size-Selective Purification System (Promega). Libraries were prepared with the SMRTbell Template Prep Kit 2.0 (PacBio) and sequenced on the PacBio Sequel I platform. Analysis was performed using the Iso-Seq Analysis in SMRT Link. Briefly, circular consensus sequences (CCS) reads were generated from the sequenced reads and primer sequences, and the poly(A) tail and concatemers were removed. The reads were then clustered, and a consensus sequence was generated for each read cluster. The polished reads were mapped to a reference sequence and visualized using the Integrated Genome Viewer.
